# *Pseudoclavibacter* Otitis Media in a 3-Year-Old Boy With Pulmonary and Spinal Tuberculosis

**DOI:** 10.1097/MD.0000000000000709

**Published:** 2015-05-01

**Authors:** Satja Issaranggoon na Ayuthaya, Amornrut Leelaporn, Pattarachai Kiratisin, Peninnah Oberdorfer

**Affiliations:** From the Department of Pediatrics, Faculty of Medicine, Chiang Mai University, Chiang Mai (SInA, PO); and Department of Microbiology, Faculty of Medicine, Siriraj Hospital, Mahidol University, Bangkoknoi, Bangkok, Thailand (AL, PK).

## Abstract

*Pseudoclavibacter* has rarely been documented as an etiologic agent of infection in humans. We presented the first case report of *Pseudoclavibacter* otitis media in a boy with pulmonary and spinal tuberculosis.

A 3-year-old boy was referred to our hospital due to prolonged fever and progressive paraplegia for 3 months. He had yellowish discharge from both ear canals. The pleural fluid culture was positive for *Mycobacterium tuberculosis*. The discharge from both ears culture yielded yellow colonies of gram-positive bacilli with branching. This organism was positive for modified acid-fast bacilli stain but negative for acid-fast bacilli stain. Biochemical characteristics of this isolate were positive for catalase test but negative for oxidase, nitrate, esculin, and sugar utilization tests. The organism was further subjected to be identified by 16S ribosomal deoxyribonucleic acid gene sequencing. The result yielded *Pseudoclavibacter* species (99.4% identical), which could be most likely a potential pathogen in immunocompromised host like this patient. He responded well with intravenous trimetroprim-sulfamethoxazole for 6 weeks.

This is the first case report of *Pseudoclavibacter* otitis media in children, and this case could emphasize *Pseudoclavibacter* species as a potential pathogen in immunocompromised host.

## INTRODUCTION

The bacteria in genus *Pseudoclavibacter*, family *Microbacteriaceae*, and class *Actinobacteria* is aerobic, nonmotile, oxidase positive but catalase negative gram-positive bacilli. In May 2004, Manaia et al^1^ reported that because of distinctive phenotypic characteristics of bacterium *Brevibacterium helvolum* DSM 20419 from 16S rDNA sequence analysis, they proposed that this strain should be reclassified as novel genus and species, with *Pseudoclavibacter helvolus*. In September 2004, Lin et al^2^ also reported that this strain DSM 20419 should be classified into novel genus and species, with *Zimmermanella helvola*, and they also proposed 3 novel species *Zimmermannella alba*, *Zimmermannella bifida*, and *Zimmermannella faecalis*. Because the name of this new genus was published by Manaia et al before by Lin et al, the strain DSM 20419 should be reclassified as *P helvolus*, and the 3 novel species *Z alba*, *Z bifida*, and *Z faecalis* should be reclassified as “*Pseudoclavibacter alba*”, “*Pseudoclavibacter bifida*”, and “*Pseudoclavibacter faecalis*”. Later, there were few publications of the isolation of novel species in genus *Pseudoclavibacter* from the environment like soil (*Pseudoclavibacter soli*) and sludge (*Pseudoclavibacter chungangensis*, *Pseudoclavibacter caeni*).^3–5^

*Pseudoclavibacter* has rarely been documented as an etiologic agent of infection in humans. There were 2 case reports in immunocompromised adults. The first case report by Lemaitre et al in 2011 was an 81-year-old French man who had subcutaneous infection despite antibiotic treatment combining clindamycin and metronidazole. A subcutaneous swab showed numerous polymorphonuclear cells, a few gram-positive cocci, gram-negative cocci, and gram-positive rods. Further investigation of gram-positive rods with gene sequence and mass spectrometry showed 99% sequence similarity with *Pseudoclavibacter* spp. and unique peptide profile of *Pseudoclavibacter* spp. The authors concluded that *Pseudoclavibacter*-like organism was identifiable in cutaneous and subcutaneous infections in humans.^6^

Later, in 2013 Oyaert et al reported the second case of *Pseudoclavibacter* infection in an 86-year-old man who had underlying chronic obstructive pulmonary disease (COPD) and received inhaled glucocorticoids and long-acting bronchodilators. He had suspected community-acquired pneumonia, and 2 aerobic blood culture bottles taken from peripheral and central line before intravenous antibiotic treatment with ceftriaxone initiation showed gram-positive rods. Further bacteriological investigation showed nonmotile, alkaline phosphatase-positive, catalase-positive, and oxidase-negative rods. The isolate grew on blood and chocolate agar after 2 days at 37°C in air supplemented with 5% carbondioxide (CO_2_). Finally the strain was tested, and 16S ribosomal deoxyribonucleic acid (*rDNA*) gene sequence exhibited 99.5% sequence similarity with *P bifida*. The authors concluded that they found the first *P bifida* septicemia in an immunocompromised COPD patient with bilateral pneumonia.^7^

However, there has never been any case report of *Pseudoclavibacter* infection in children. We presented the first case report of *Pseudoclavibacter* otitis media in a 3-year-old boy with pulmonary and spinal tuberculosis.

## PRESENTING CONCERNS

A 3-year-old boy from Myanmar was referred from district hospital to Chiang Mai University Hospital due to prolonged fever and progressive paraplegia. He had low-grade fever and weakness of both legs for 3 months but no fecal or urinary incontinence. He had good orientation and was able to speak Thai fluently. He also had yellowish discharge from both ear canals.

## CLINICAL FINDINGS

The PE revealed temperature of 38.4°C, respiratory rate of 60 breaths/min, pulse rate of 154 beats/min, yellowish purulent discharge from both ears, decreased breath sound of right lung, and kyphosis of upper back. The neurological examination showed good consciousness, grade III weakness of both lower extremities, hyperreflexia both lower extremities, and positive knee and ankle clonus both sides.

## TIMELINE

Timeline for *Pseudoclavibacter* otitis media in a 3-year-old boy with pulmonary and spinal tuberculosis was shown in Table 1.

**TABLE 1:**
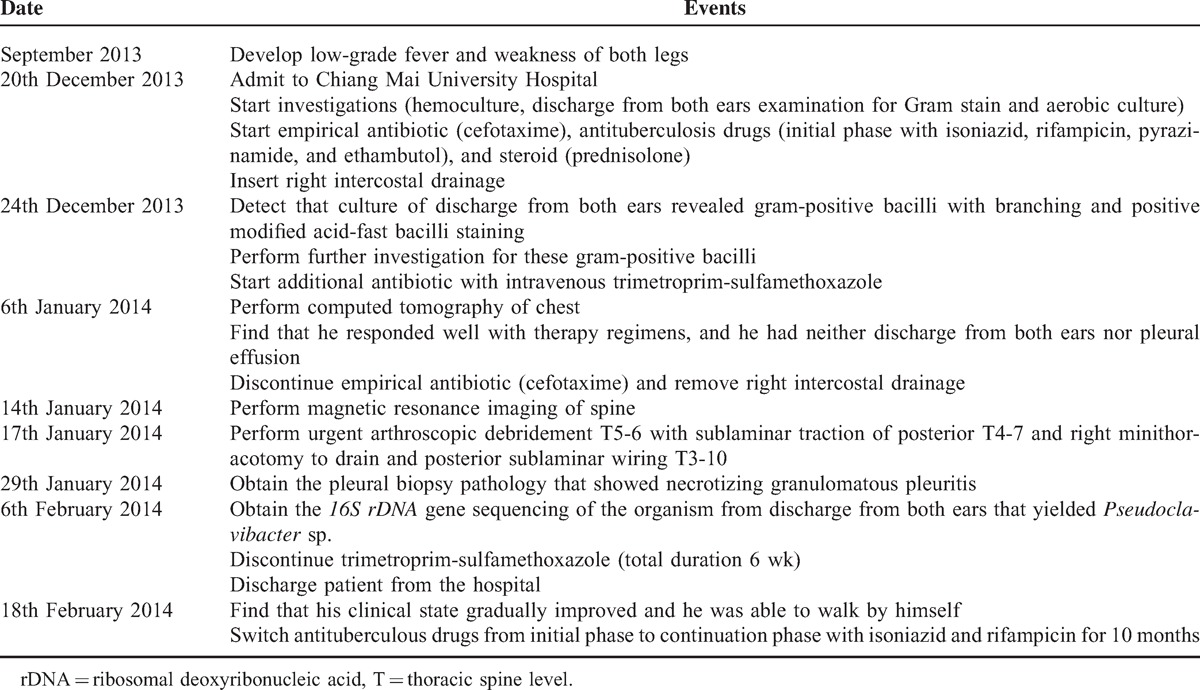
Timeline for *Pseudoclavibacter* Otitis Media in a 3-Year-Old Boy With Pulmonary and Spinal Tuberculosis

## DIAGNOSTIC FOCUS AND ASSESSMENT

**FIGURE 1:**
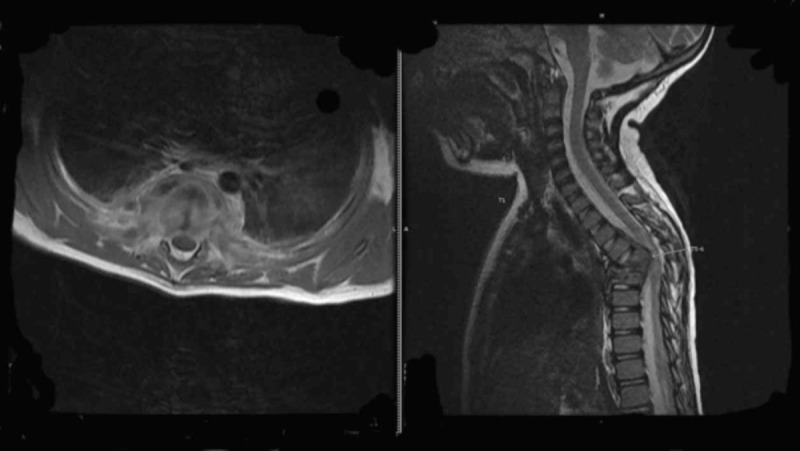
The spinal magnetic resonance imaging showed T5-6 infective spondylitis with pre/paraverteral enhancing soft tissues and abscess formation causing spinal cord compression and cord edema at the same level.

The complete blood count showed hemoglobin of 8.1 g/dL, hematocrit of 26.7%, white blood cells of 10,400 cell/mm^3^ (neutrophil 64% and lymphocyte 22%), and platelet of 519,000/mm^3^. The chest computerized tomography showed pleural thickening of right hemithorax with small loculated right pleural effusion. The spinal magnetic resonance imaging showed T5-6 infective spondylitis with pre-/paraverteral enhancing soft tissues and abscess formation causing spinal cord compression and cord edema at the same level (Figure 1). He received urgency surgical operation for arthroscopic debridement T5-6 with sublaminar traction of posterior T4-7 and for video-assisted thoracoscopic surgery. The pleura biopsy revealed granulomatous pleuritis, and pleural fluid culture was positive for *Mycobacterium tuberculosis*.

Direct microscopic examination of purulent discharge from both ears showed numerous polymorphonuclear cells and many gram-positive rods. The discharge from both ears was also taken for aerobic culture. After inoculation on blood and chocolate agar, incubated at 35°C in 5% CO_2_ incubator for 2 days, the plates yielded yellow colonies of gram-positive bacilli with branching. This organism was positive for modified acid-fast bacilli stain but negative for acid-fast bacilli stain. Biochemical characteristics of this isolate were positive for catalase test but negative for oxidase, nitrate, esculin, and sugar (glucose, maltose, lactose, raffinose, and inositol) utilization tests. This bacterium could grow in 0% sodium chloride (NaCl) medium but not in 7% NaCl. The organism was further subjected to be identified by *16S rDNA* gene sequencing. The result yielded *Pseudoclavibacter* species (99.4%).

## THERAPEUTIC FOCUS AND ASSESSMENT

We treated this patient with intravenous trimetroprim-sulfamethoxazole (20 mg of trimetroprim/kg/day intravenous every 6 hour) for 6 weeks. We also treated him with antituberculous drugs (initial phase with isoniazid, rifampicin, pyrazinamide, and ethambutol for 2 months and continuation phase with isoniazid and rifampicin for 10 months), oral prednisolone (1 mg/kg/day for 1 month) due to pleural effusion, and empirical antibiotic for otitis media (cefotaxime 100 mg/kg/day) for 14 days.

## FOLLOW-UP AND OUTCOMES

He responded well with these therapy regimens. He had neither discharge from both ears nor pleural effusion within 2 weeks after treatment. He also had no adverse events from treatments and his clinical state gradually improved. He was able to walk by himself at the second month of the treatment.

## DISCUSSION

In our patient, many gram-positive rods were observed from the discharge from both ears of 3-year-old boy with pulmonary and spinal tuberculosis. The further *16S rDNA* gene sequencing could identify this organism as *Pseudoclavibacter* sp. So we assumed that this is the first case report of *Pseudoclavibacter* otitis media in children, and this case could emphasize *Pseudoclavibacter* species as a potential pathogen in immunocompromised host. These organisms could be successfully treated with intravenous trimetroprim-sulfamethoxazole for 6 weeks like this patient.

## INFORMED CONSENT

Informed consent has been provided from the patient's parent for the publication of this case report.
